# Protein corona of airborne nanoscale PM2.5 induces aberrant proliferation of human lung fibroblasts based on a 3D organotypic culture

**DOI:** 10.1038/s41598-018-20445-7

**Published:** 2018-01-31

**Authors:** Yan Li, Pengcheng Wang, Chuanlin Hu, Kun Wang, Qing Chang, Lieju Liu, Zhenggang Han, Yang Shao, Ying Zhai, Zhengyu Zuo, Michael Mak, Zhiyong Gong, Yang Wu

**Affiliations:** 1 Key Laboratory for Deep Processing of Major Grain and Oil (Wuhan Polytechnic University), Ministry of Education, College of Food Science and Engineering, Wuhan Polytechnic University, Wuhan, 430023 P. R. China; 2 Hubei Key Laboratory for Processing and Transformation of Agricultural Products (Wuhan Polytechnic University), College of Food Science and Engineering, Wuhan Polytechnic University, Wuhan, 430023 P. R. China; 3 Department of Biomedical Engineering, School of Engineering & Applied Science, Yale University, New Haven, 06520 USA; 4 Division of Biostatistics, Department of Population Health, New York University, New York, 10016 USA; 5 Department of Chemical and Biomolecular Engineering, Hong Kong University of Science & Technology, Clear Water Bay, Kowloon, Hong Kong; 6 State Key Laboratory of Silicate Materials for Architectures, Wuhan University of Technology, Wuhan, 430070 P. R. China; 7 School of Biology and Pharmaceutical Engineering, Wuhan Polytechnic University, Wuhan, 430023 P. R. China

## Abstract

Exposure to PM2.5 has become one of the most important factors affecting public health in the world. Both clinical and research studies have suggested that PM2.5 inhalation is associated with impaired lung function. In this study, material characterization identified the existence of nanoscale particulate matter (NPM) in airborne PM2.5 samples. When coming into contact with protein-rich fluids, the NPM becomes covered by a protein layer that forms a “protein corona”. Based on a 3D organotypic cell culture, the protein corona was shown to mitigate NPM cytotoxicity and further stimulate the proliferation of human lung fibroblasts (HLFs). ROS-activated alpha-smooth muscle actin (α-SMA) is considered to be one of the proliferation pathways. In this research, 3D cell cultures exhibited more tissue-like properties compared with the growth in 2D models. Animal models have been widely used in toxicological research. However, species differences make it impossible to directly translate discoveries from animals to humans. In this research, the 3D HLF model could partly simulate the biological responses of NPM-protein corona-induced aberrant HLF proliferation in the human lung. Our 3D cellular results provide auxiliary support for an animal model in research on PM2.5-induced impaired lung function, particularly in lung fibrosis.

## Introduction

Air pollution is one of the most important public health issues in the world^1^. Airborne particulate matter 2.5 (PM2.5, diameter ≤ 2.5 μm) is considered to be one of the most hazardous factors^2^. Epidemiological investigations suggest that human lung fibrosis is associated with PM2.5, but the functional role of PM2.5 remains unclear^3,4^. The majority of existing research has generally focused on particles whose diameter is less than 2.5 microns^5^. However, we should pay more attention to airborne nanoscale particulate matter (NPM)^6^ because NPM is more likely than larger particles to be produced by fuel combustion or formed by secondary reactions. In addition, NPM can remain in the atmosphere for weeks and can thus be transported over longer distances in the atmosphere compared to larger particles^7^.

NPM is expected to exhibit particulate physiological characteristics due to the tiny size and special structure of its particles. Once inhaled, they may have a high probability of being deposited in the lung^8^. Due to the large surface areas of such particles, NPM could exhibit extensive surface activity and absorb protein-rich fluids *in vivo*. The absorbed protein forms a ‘crown’, which is called a protein corona^9^. Researchers report that the protein corona may affect cellular interactions^10^, cytokine expression^11^ and protein function^12^, which further disturbs regular metabolism and organ function^13^. Therefore, a thorough understanding of the protein corona is useful when investigating the involvement of PM2.5 in pulmonary dysfunction.

Animal models have been widely used in toxicological research of PM2.5, and they continue to aid our understanding of various diseases. However, we need to recognize that there are potential differences between an animal model and humans^14^. Even animals very similar to humans, such as primates, have failed to predict what will occur in humans. Therefore, it is impossible to directly translate discoveries from animals to humans. The human organotypic 3D cell model provides auxiliary support for animal models in toxicology research and could partly simulate the physiological response of humans^15^. Compared to traditional 2D cell cultures (cells cultured on a flat surface), 3D cell cultures provide a more physiologically tissue-like environment for cells. This environment allows cells *in vitro* to grow in all directions, which is similar to how they would grow *in vivo*. 3D cell models are more robust and provide more comprehensive and relevant biological information than that obtained from 2D models^16–18^.

Various methods have been developed to meet the growing demand for 3D cell cultures. Scaffold-based 3D culture models have generally been adopted by researchers. 3D scaffolds can be manufactured from a range of natural and synthetic materials. Naturally derived materials are mainly based on extracellular matrix (ECM) components (e.g., collagen and fibrin), which can better mimic the biophysical and biochemical complexity of tissues than synthetic materials^19^. These materials are biocompatible, biodegradable and contain cell adhesion sites. More recently, fibrin scaffolds, produced by polymerizing the protein fibrinogen obtained from plasma, have been widely investigated for a variety of tissue engineering applications^20,21^. These include the study of fibroblast activation and proliferation based on fibroblast-fibrin matrix culture models^22,23^. These studies investigated the relationship between fibrinogen concentrations, cell proliferation, and stiffness in 3D fibrin matrices. All results indicated that the fibroblast-fibrin matrix culture facilitated the analysis of cell physiology under conditions that more closely resemble an *in vivo*-like environment compared to conventional 2D cell cultures. It is believed that fibrin matrix 3D culture models will be a useful platform to investigate PM2.5 involvement in pulmonary dysfunction.

Abnormal proliferation and activation of lung fibroblasts are known to contribute to the initiation and progression of idiopathic pulmonary fibrosis. In this study, a 3D HLF culture was established in a microfluidic chip. The combination of a fibrin hydrogel with a microfluidic device has been used for an HLF 3D-organotypic culture. This established model *in vitro* is proposed to mimic some of the physiological features of an HLF in the human lung. Meanwhile, the morphology, components and size of the NPM were analysed. We hypothesized that NPM exposed to serum would be covered by a protein crown called the protein corona. We also characterized the physicochemical properties of the NPM-protein corona in this research. Taking into account established exposure levels, we used different NPM-protein coronas (5, 10 and 20 μg/ml NPM) for the 3D-HLF exposures. We observed and analysed the biological responses of the 3D-HLF under *in vivo*-like conditions. These responses included cell proliferation, oxidative stress biomarkers (ROS, MDA, GSH, 8-OH-dG), and alpha-smooth muscle actin (α-SMA) expression. The objective was to investigate the potential mechanism of NPM-protein corona-induced HLF proliferation under three-dimensional conditions, which is similar to how they would grow *in vivo*. This study is useful for understanding the underlying mechanisms of PM2.5 involvement in human lung fibrosis.

## Results

### Physicochemical characteristics of NPM

**Figure 1:**
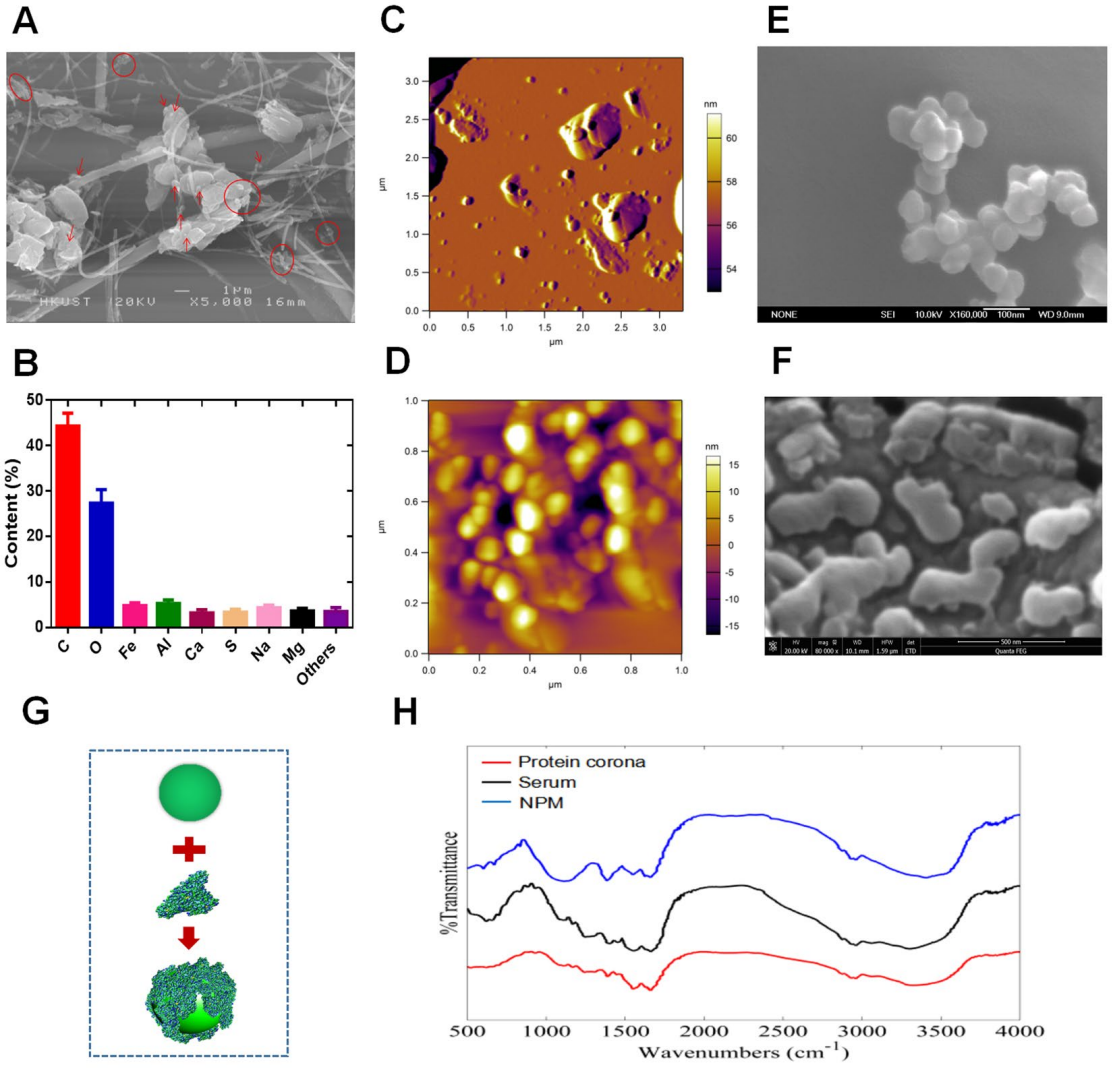
Physicochemical characterization of airborne particulate matter. (**A**) SEM images of airborne PM2.5 (nanoscale PM2.5 is marked in red). (**B**) Chemical element analysis of PM2.5 by EDX analysis. (**C**) AFM image of nanoscale PM2.5 (NPM) from air pollutant samples (scale 3.5 μm). (**D**) AFM image of airborne NPM from air pollutant samples (scale 1 μm). (**E**) SEM image of airborne NPM from air pollutant samples. (**F**) SEM image of an NPM-protein corona. (**G**). Schematic diagram of the biological interaction between NPM and protein. (**H**). FTIR spectra of NPM, serum and NPM-protein corona.

The initial air samples were characterized using scanning electron microscopy (SEM). SEM images show that the PM2.5 particles had adhered to the filament network. The collected particle diameter was less than 2.5 microns. In addition, NPM was also observed from collected airborne samples (Fig. 1A). The main chemical element distributions of the particles were analysed using energy-dispersive X-ray (EDX) spectroscopy. EDX analysis showed that PM2.5 consisted of up to 43% carbon and up to 27% oxygen, with smaller contributions of iron, aluminium, calcium, sulfur, sodium and magnesium (Fig. 1B). Of all the observed particles, we focused on only particles with a nanoscale aerodynamic diameter. The three-dimensional morphology of airborne nanoscale particles was characterized by atomic force microscopy (AFM). AFM can identify particles of less than 1 micron in the original air pollutant samples (Fig. 1C). The AFM image clearly showed that most of the particles were in the ten to hundreds of nanometres range (Fig. 1D). The morphology of every single NPM particle was further revealed by AFM. Figure 1D gives the size distribution and height of a single dispersed NPM.

### Biological interaction of NPM and protein

We studied the interaction between NPM and protein in this research. The morphology of a single NPM particle (diameter less than 100 nanometres) was demonstrated using SEM (Fig. 1E). SEM also shows the “protein corona” formed after NPM reacted with serum. Figure 1F shows that the NPM particle was surrounded by a protein cloud. The NPM-protein corona complex appeared to be larger than the original diameter of the NPM particle. The average diameter of these complexes ranged from 100 to 200 nanometres. Based on a comparison to the average diameter of a naked NPM particle (~50 nm), the dimensional increment can be attributed to the “protein coat” (Fig. 1G). To better confirm the interaction between NPM and protein, an FTIR analysis was performed to verify the protein corona. Prior to the FTIR analysis, samples were centrifuged and washed with DI water to remove any unabsorbed proteins. As shown in Fig. 1H, the FTIR spectrum of serum consists of several characteristic peaks (~1200–1500 cm^−1^). The FTIR spectrum of the NPM-protein corona is similar to that of the serum. However, NPM produced a different spectrum from the NPM-protein corona and serum spectra. The FTIR results therefore further confirmed that NPM was covered by a protein crown.

### 3D organotypic culture of HLFs

**Figure 2:**
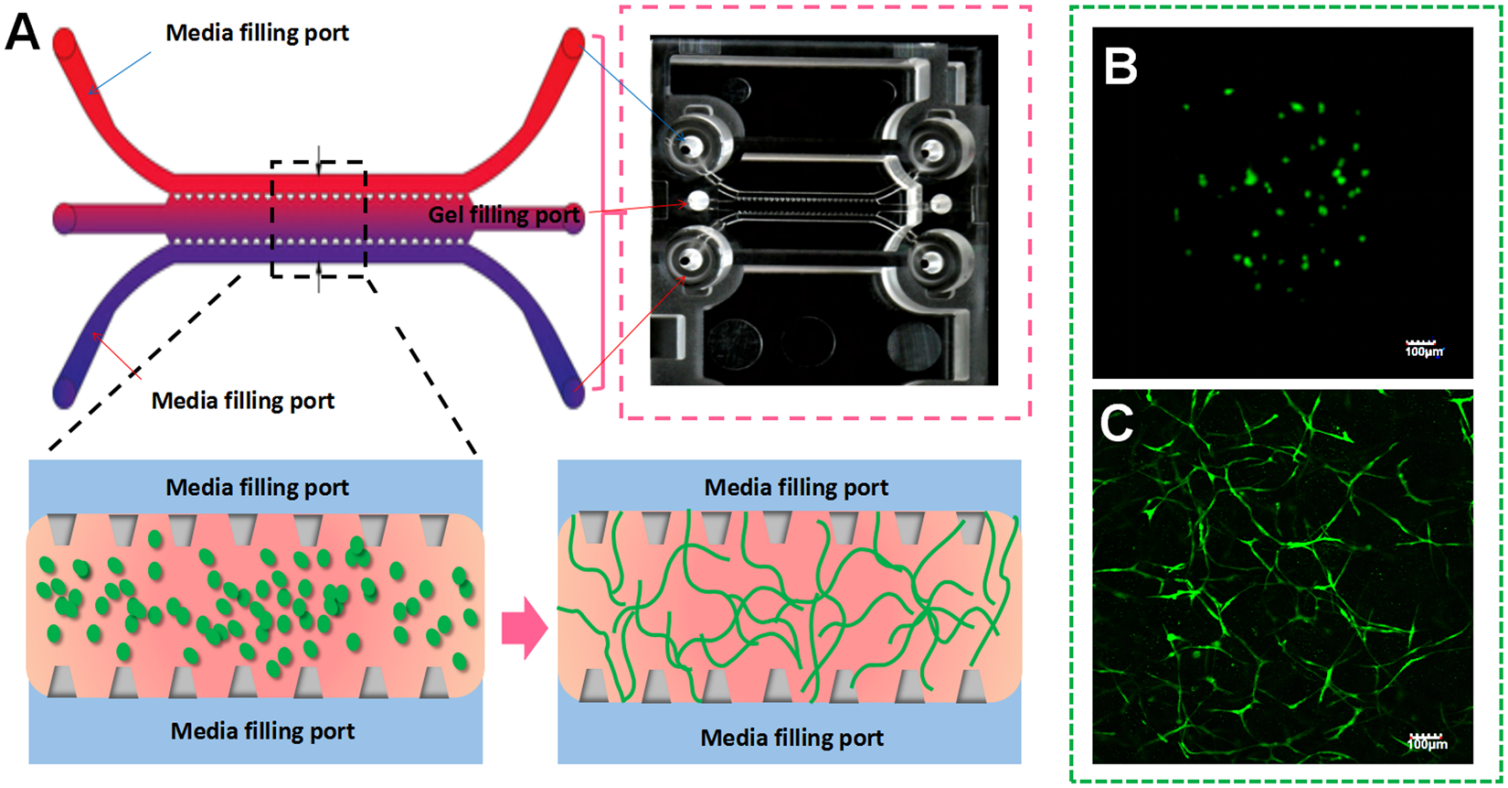
3D organotypic culture of human lung fibroblasts (HLFs). (**A**) Schematic diagram of cell gelation in a microfluidic chip. (**B**) Confocal microscope image of HLFs just after gelation in a microfluidic chip (magnification = ×10). (**C**) Confocal microscope image of 3D-HLF after three days of culture (magnification = ×10).

**Figure 3:**
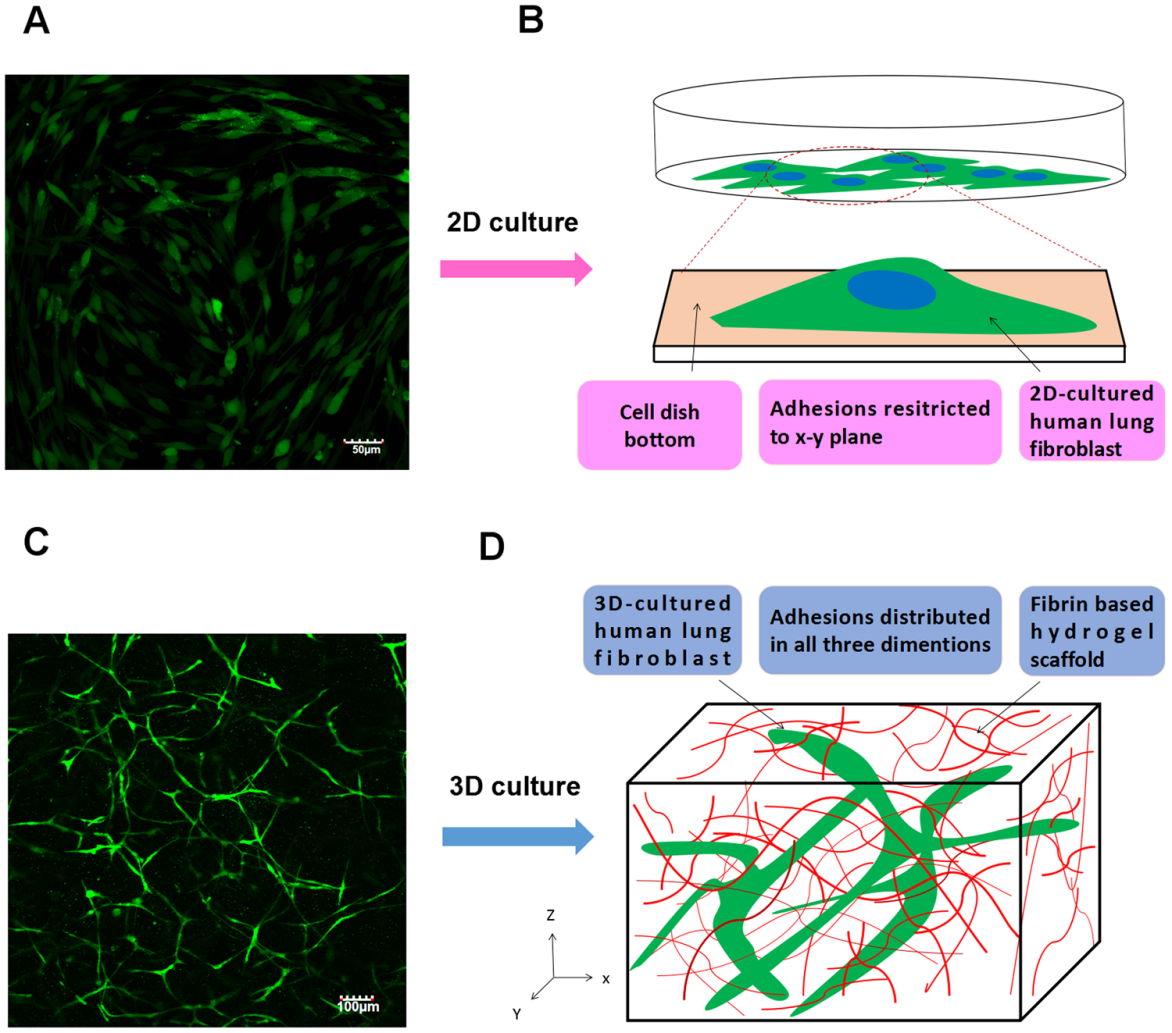
2D versus 3D cultures of human lung fibroblasts (HLFs). (**A**) Confocal microscope image of 2D-cultured HLFs (magnification = ×10). (**B**) Schematic diagram of a 2D culture: HLFs attached to the cell dish bottom. (**C**) Confocal microscope image of 3D-cultured HLFs (magnification = ×10). (**D**) Schematic diagram of a 3D culture: HLFs grow in fibrin-based scaffolds.

**Figure 4:**
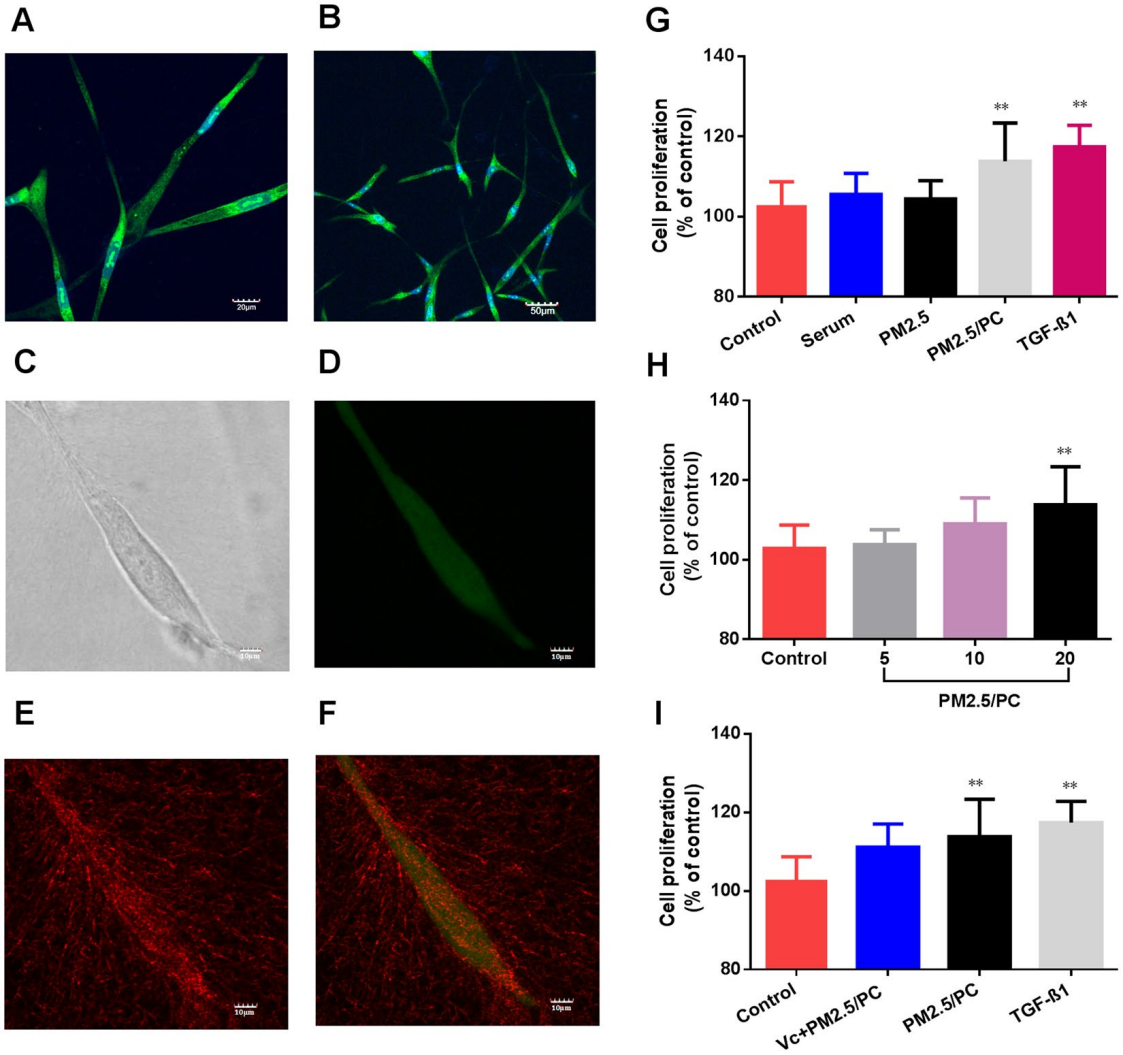
3D organotypic culture of human lung fibroblasts (HLFs). (**A**) Confocal image of fluorochrome-stained live cells and nuclei (magnification = ×40). (**B**) Confocal image of fluorochrome-stained live cells and the nucleus (magnification = ×20). (**C**) High magnification of the confocal image of a single HLF (magnification = ×40, zoom in factor = 2.5). (**D**) High magnification of the confocal image of a fluorochrome-stained single HLF (magnification = ×40, zoom in factor = 2.5). (**E**) High magnification of the confocal image of a fibrin-based scaffold (magnification = ×40, zoom in factor = 2.5). (**F**) Merged confocal image of a fibrin matrix covered by HLFs (magnification = ×40, zoom in factor = 2.5). Cell viability of 3D-cultured HLFs by stimulation of (**G**) control, serum, nanoscale PM2.5 (20 μg/ml), nanoscale PM2.5 (20 μg/ml)-protein corona (PC), and TGF-β1(2 ng/ml); (**H**) control and nanoscale PM2.5 (5, 10 and 20 μg/ml)-protein corona; (**I**) control and nanoscale PM2.5-protein corona/V_C_ (vitamin C-0.1 mg/ml, nanoscale PM2.5–20 μg/ml); and nanoscale PM2.5 (20 μg/ml)-protein corona and TGF-β1 (2 ng/ml) (**P < 0.01, compared with the control).

In this research, fibrin gel was applied to generate scaffolds for the 3D organotypic culture of HLFs. The advantages of fibrin gel compared to synthetic polymeric materials are that fibrin gel is nontoxic, with controllable degradation and excellent biocompatibility. Isolated HLFs were first suspended in the scaffold precursor solution of fibrinogen and thrombin. Then, the mixture was delivered into a microfluidic chip (Fig. 2A) and cultured for 1 hour to complete the gelation (Fig. 2B). After gelation, the HLFs were growing and extending in the fibrin based on 3D scaffolds (Fig. 2C). Cell development and status were monitored using a confocal microscope. Compared to a 2D culture (Fig. 3A,B), the cells clearly showed very different morphologies in the 3D conditions. 3D-culturd HLFs had a thinner and more fibre-like morphology (Fig. 3C,D), while cells in 2D culture exhibited a more widely spread morphology. After being cultured for three days, these fibre-like cells formed 3D networks (Fig. 3C), which were similar to the constructs of *in vivo* organs. More details of the cell growth were further observed using a high-magnification confocal microscope. Fluorescent dye was applied to identify live cells and the nucleus. HLFs emit green fluorescence, which indicates live cells. Live cells with a clear and intact cell nucleus were observed using a confocal microscope with high magnification (Fig. 4A,B). High-magnification images of HLFs (Fig. 4C,D) and the fibrin-matrix (Fig. 4E) were also captured. The images further demonstrate the cell growth and extension in the fibrin scaffolds. The fibrin-matrix was stretched by cell movement (Fig. 4E). This clearly demonstrates that HLFs were stretching well in the fibrin-based 3D culture model (Fig. 4F).

### NPM-protein corona stimulates the proliferation of 3D-cultured HLFs

3D-cultured HLFs were initially exposed to different groups of NPM-protein corona preparations (5, 10 and 20 μg/ml NPM) for three days. The results demonstrated that the NPM-protein corona potentially promoted the cell growth of 3D-cultured HLFs. Cell numbers increased in a dose-dependent manner (Fig. 4H). The cell viabilities were 103%, 109% and 113%, respectively. The NPM-protein corona (20 μg/ml NPM) significantly promoted an increase in HLFs (p < 0.01) compared to the control group. To verify the biological function of the protein corona, pure serum and NPM (20 μg/ml) were also tested. The cell viabilities were 105% and 104%, respectively (Fig. 4G). In addition, the group of vitamin C (0.1 mg/ml)-NPM-protein corona (NPM is 20 μg/ml) was also exposed to 3D-cultured HLFs. Cell viability declined compared to that with the NPM-protein corona (20 μg/ml NPM) (Fig. 4I). Finally, TGF-β1 was also shown to promote HLF proliferation in the 3D model (Fig. 4I).

### NPM-protein corona mediates oxidative stress

**Figure 5:**
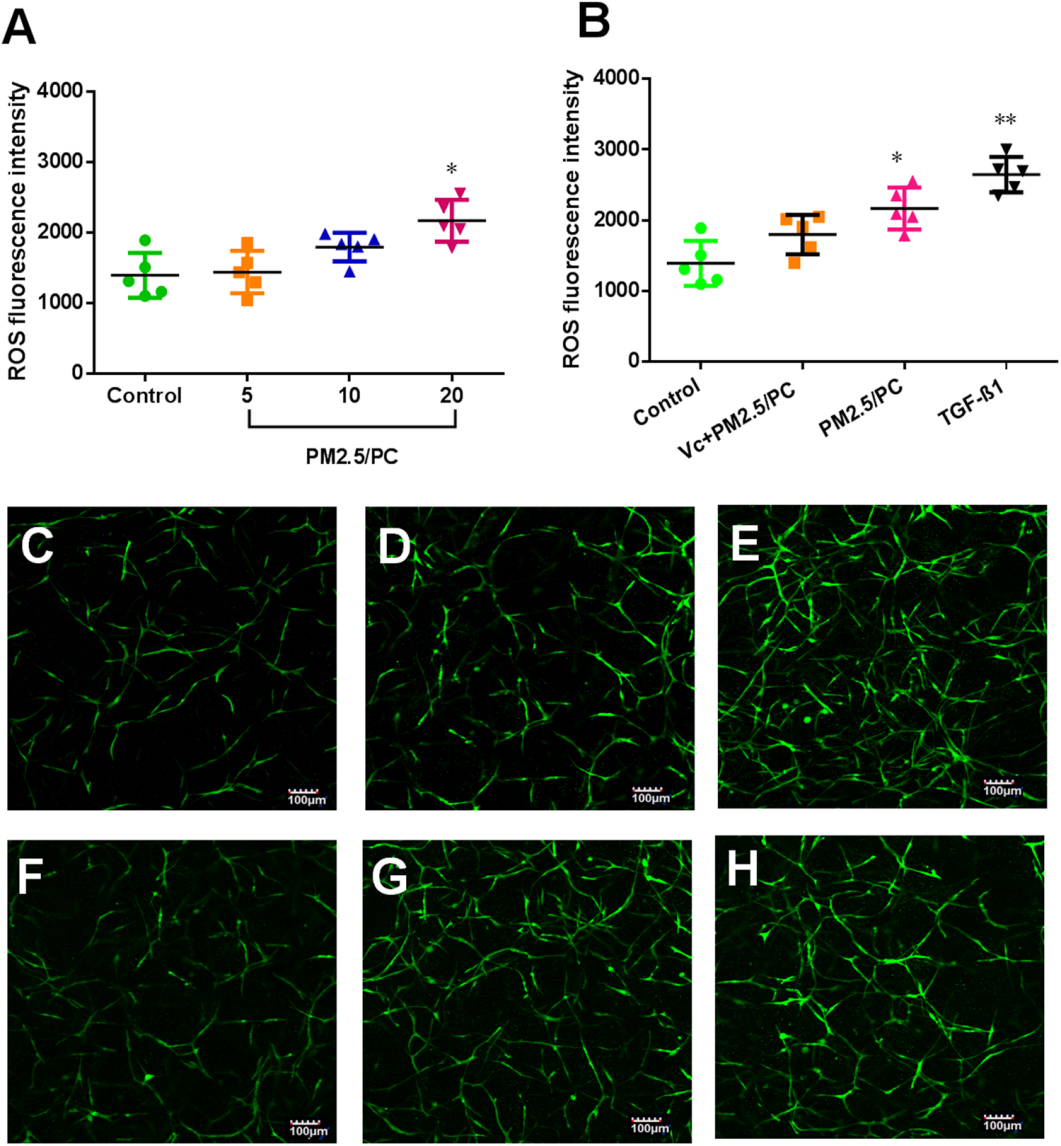
ROS fluorescence intensity. (**A**) Nanoscale PM2.5 (5, 10 and 20 μg/ml)-protein corona (PC). (**B**) Nanoscale PM2.5-protein corona/V_C_ (vitamin C-0.1 mg/ml, nanoscale PM2.5–20 μg/ml), nanoscale PM2.5 (20 μg/ml)-protein corona, and TGF-β1 (2 ng/ml) (*P < 0.05, **P < 0.01, compared with the control). Images of fluorochrome-labelled ROS in 3D-cultured HLFs (magnification = ×10): (**C**) control (with HLF medium), (**D**) nanoscale PM2.5-protein corona/V_C_ (vitamin C-0.1 mg/ml, nanoscale PM2.5–20 μg/ml), (**E**) TGF-β1 (2 ng/ml), and (F-H) nanoscale PM2.5 (5, 10 and 20 μg/ml)-protein corona.

**Figure 6:**
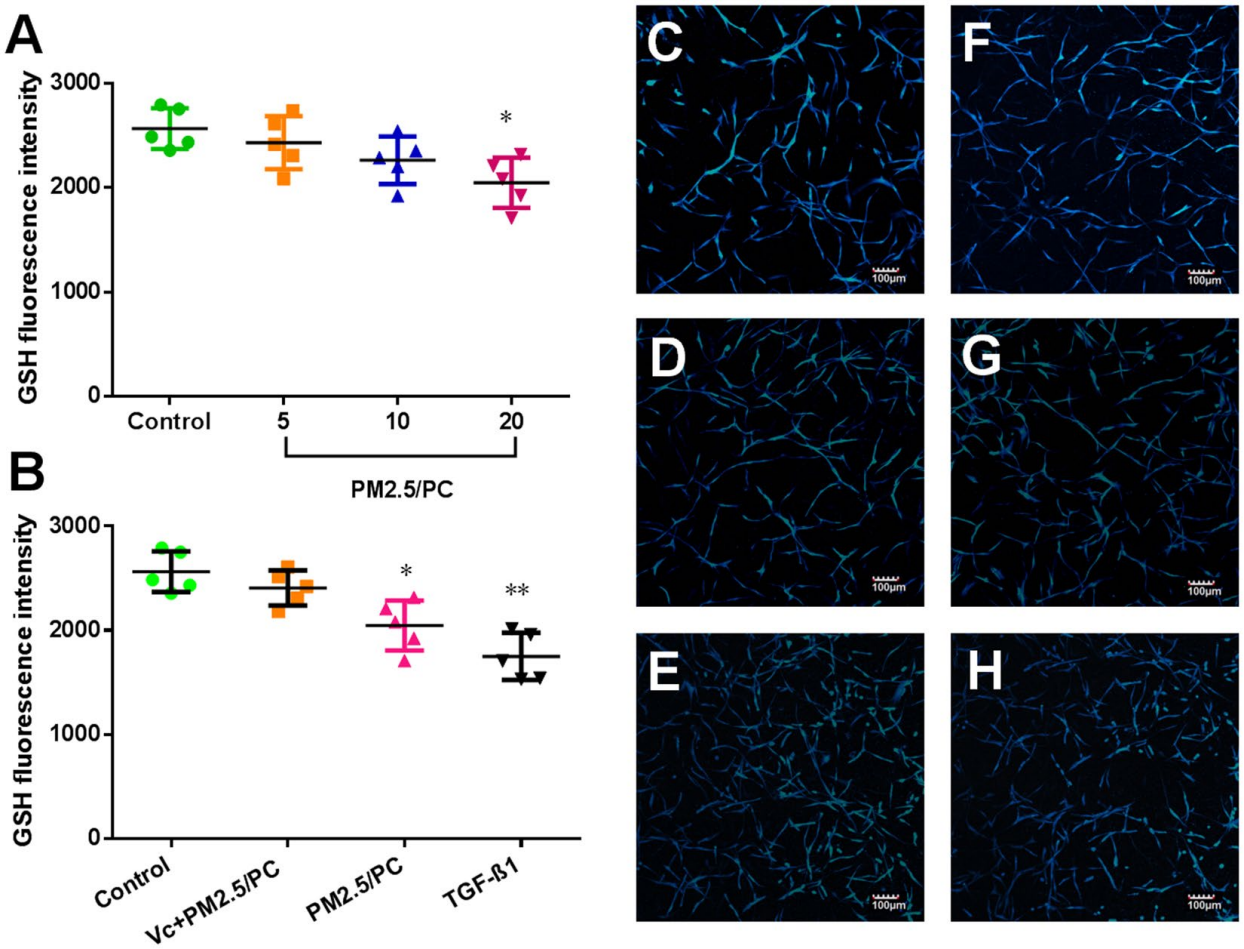
GSH fluorescence intensity. (**A**) Nanoscale PM2.5 (5, 10 and 20 μg/ml)-protein corona (PC). (**B**) Nanoscale PM2.5-protein corona/V_C_ (vitamin C-0.1 mg/ml, nanoscale PM2.5–20 μg/ml), nanoscale PM2.5 (20 μg/ml)-protein corona, and TGF-β1 (2 ng/ml) (*P < 0.05, **P < 0.01, compared with the control). Images of fluorochrome-labelled GSH in 3D-cultured HLFs (magnification = ×10): (**C**) control (with HLF medium), (**D**) nanoscale PM2.5-protein corona/V_C_ (vitamin C-0.1 mg/ml, nanoscale PM2.5–20 μg/ml), (**E**) TGF-β1 (2 ng/ml), and (**F**–**H**) nanoscale PM2.5 (5, 10 and 20 μg/ml)-protein corona.

**Figure 7:**
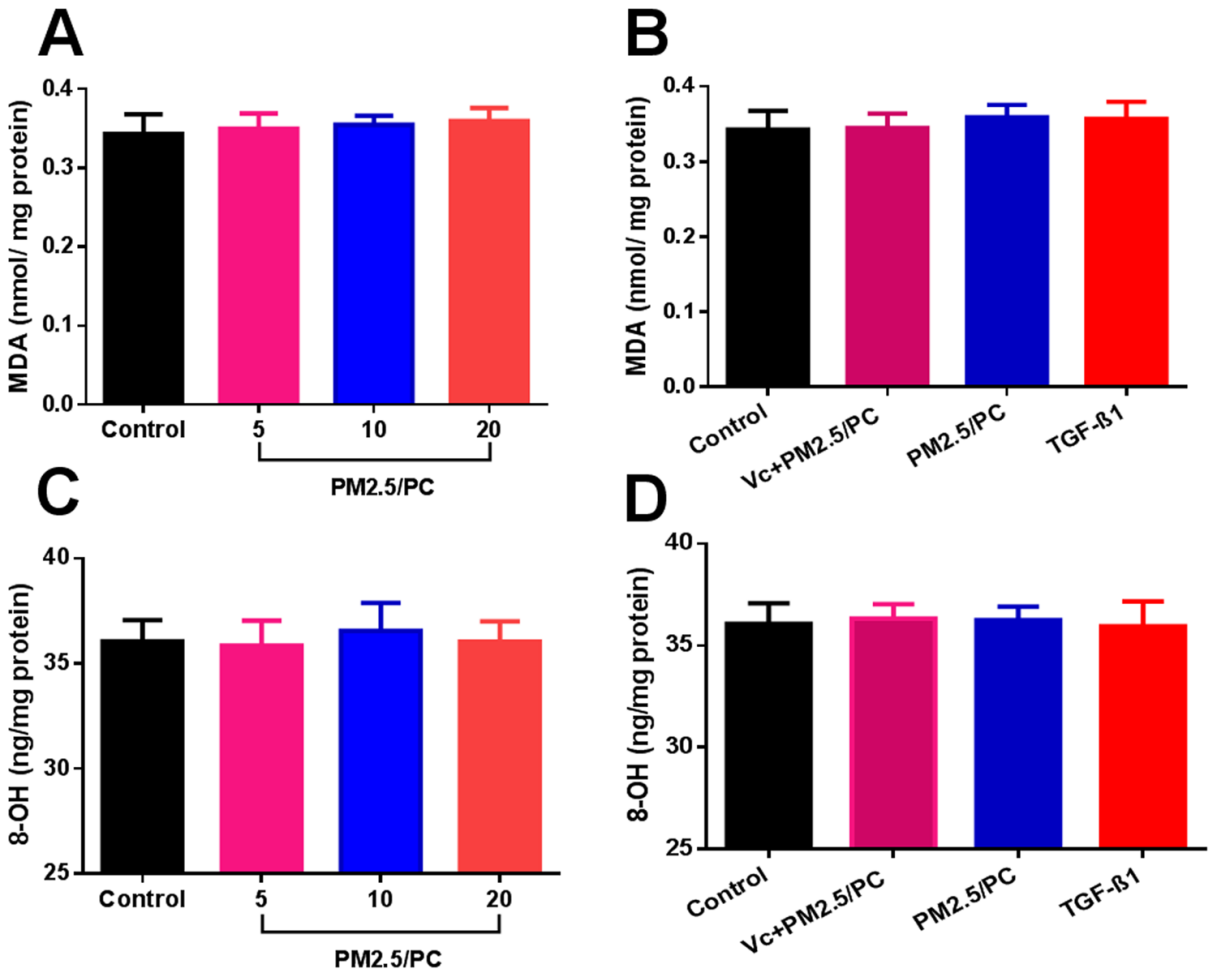
Intracellular MDA of 3D-cultured human lung fibroblasts (HLFs). (**A**) Nanoscale PM2.5 (5, 10 and 20 μg/ml)-protein corona (PC) and (**B**) nanoscale PM2.5-protein corona/V_C_ (vitamin C-0.1 mg/ml, nanoscale PM2.5–20 μg/ml); nanoscale PM2.5 (20 μg/ml)-protein corona; TGF-β1 (2 ng/ml). Intracellular 8-OH-dG of 3D-cultured human lung fibroblasts (HLFs). (**C**) Nanoscale PM2.5 (5, 10 and 20 μg/ml)-protein corona. (**D**) Nanoscale PM2.5-protein corona/V_C_ (vitamin C-0.1 mg/ml, nanoscale PM2.5–20 μg/ml); nanoscale PM2.5 (20 μg/ml)-protein corona; TGF-β1 (2 ng/ml) (**P < 0.01, compared with the control).

Specific fluorescent agents were used to identify the cellular expression of ROS and GSH. Figure 5A shows a positive ROS increase in relation to the NPM concentration. An antioxidant (vitamin C) effectively repressed ROS generation (Fig. 5B). TGF-β1 promoted ROS, as has been reported in the literature (Fig. 5B). Accordingly, GSH depletion was detected in all groups of NPM-protein corona preparations and TGF-β1 (Fig. 6A,B). Vitamin C effectively suppressed GSH depletion (Fig. 6B). The specific fluorescence images of ROS and GSH further confirmed these results (Fig. 5C–H and Fig. 6C–H). The NPM-protein corona stimulated ROS generation but induced no MDA increase (Fig. 7A). The NPM-protein corona also induced no variation in 8-OH-dG levels in the 3D-cultured HLFs (Fig. 7C). Moreover, the antioxidant (vitamin C) and TGF-β1 showed no change in MDA or 8-OH-dG (Fig. 7B,D). Taken together, these results demonstrate that the NPM-protein corona is expected to result in a limited increase in ROS levels. The protein corona is effectively controlled by the ROS level. However, ROS generation induced no oxidative damage.

### NPM-protein corona promotes α-SMA expression

**Figure 8:**
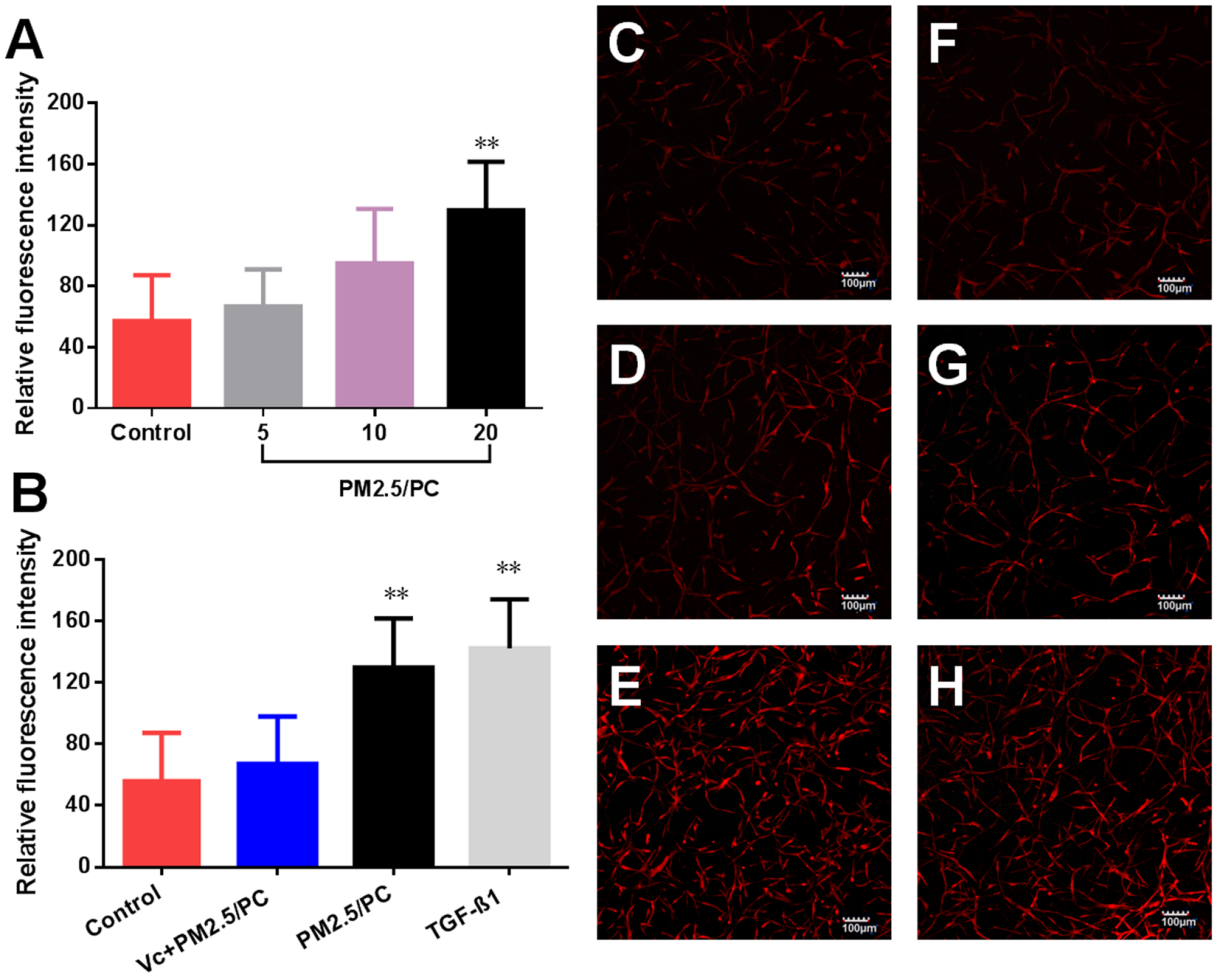
α-SMA fluorescence intensity. (**A**) Nanoscale PM2.5 (5, 10 and 20 μg/ml)-protein corona (PC). (**B**) Nanoscale PM2.5-protein corona/V_C_ (vitamin C-0.1 mg/ml, nanoscale PM2.5–20 μg/ml); nanoscale PM2.5 (20 μg/ml)-protein corona; TGF-β1 (2 ng/ml) (**P < 0.01, compared with the control); Images of fluorochrome-labelled α-SMA in 3D-cultured HLFs (magnification = ×10): (**C**) control (with HLF medium), (**D**) nanoscale PM2.5-protein corona/V_C_ (vitamin C-0.1 mg/ml, nanoscale PM2.5–20 μg/ml), (**E**) TGF-β1 (2 ng/ml), and (**F**–**H**) nanoscale PM2.5 (5, 10 and 20 μg/ml)-protein corona.

α-SMA is a key protein expressed by activated HLFs but not by quiescent fibroblasts. In this study, fluorescent agents were used to identify α-SMA expression. The results demonstrated that the NPM-protein corona stimulated HLFs to express α-SMA. α-SMA expression was positively correlated with the NPM exposure concentration (Fig. 8A). Vitamin C effectively repressed the secretion of α-SMA (Fig. 8B). TGF-β1 promoted α-SMA expression (Fig. 8B). Fluorescence images further confirmed these results (Fig. 7C–H).

## Discussion

The impact of nanoscale airborne particles on the human respiratory system is of great concern. This is because the small size facilitates the migration of these particles across any biological barrier, enabling them to enter organs *in vivo*^24,25^. The existence of these particles is recognized as a potential risk to both human health and the environment. Compared to micron-sized particles, nanoscale particles are believed to exhibit unique physicochemical properties. Due to a larger proportion of surface atoms, nanoparticles possess a higher surface energy than larger sized particles^26^. This higher surface energy enables nanoparticles to easily attract biomolecules to their surface. For example, once in contact with protein-rich fluids, a nanoparticle’s surface will be enveloped by a long-lived protein layer^27,28^. In this research, our results (SEM and FTIR) demonstrated that NPM particles were surrounded by a protein cloud to form a “protein corona”. The biological interaction between nanoparticles and proteins is based on a variety of complementary physical forces. These include van der Waal’s (VDW) interactions^29^, hydrogen bonding^30^, and electrostatic and hydrophobic interactions^31^. These physical properties may further alter the responses of the biological system. For example, the protein corona is reported to influence cellular uptake and inflammation^32^. Compared with the high nanotoxicity of naked nanoparticles, the protein corona appears to mitigate cytotoxicity^33,34^. Based on the above results, we hypothesize that the NPM-protein corona plays an essential role in PM2.5-related human pulmonary disease. However, the main mechanisms of this role are still unclear.

Epidemiological investigations have indicated that impaired lung function is associated with PM2.5 in humans^35,36^. Animal models have been widely used in scientific research on PM2.5-related lung disease^37,38^. There is, however, a growing awareness of the limitations of animal research when extrapolating the results to human beings. The failure to translate from animals to humans is likely due in part to poor homology. Currently, the investigation of human biological processes is based largely on studies of homogenous populations of cells cultured on flat substrates (2D culture). However, the 2D cell model might not faithfully capture the physiological behaviour of cells *in vivo*. Common 2D cell cultures do not adequately represent the functions of 3D tissues that have extensive cell-cell and cell-matrix interactions, as well as markedly different diffusion/transport conditions. Hence, testing cytotoxicity in 2D cultures may not accurately reflect the actual toxicity of PM2.5 in the human body. Indeed, organotypic cells primarily exist embedded within a complex and information-rich environment that contains multiple extracellular matrix (ECM) components. Modern 3D culture technology could provide such a growth microenvironment for planted cells. That is why cell types can regain their physiological form and function when embedded in a 3D culture environment. Therefore, 3D cell culture models have been introduced to bridge the gap between *in vitro* 2D cell culture and *in vivo* models^39^. Based on fibrin gelation, a human organotypic 3D culture was used in this research. In contrast to a 2D culture, HLFs cultured in a 3D model maintain a more physical structure and possess more natural 3D dimensions. The HLF network generation further demonstrated good cell communication within this established 3D culture model. In addition, fluorescence images indicated that the 3D-cultured HLFs were alive and had intact cell nuclei. High-magnification images showed that the fibrin matrix was stretched by the HLFs, further improving the cell extension in fibrin scaffolds. Based on the above results, it is clearly shown that fibrin-based 3D scaffolds provide a favourable growth environment for HLFs. HLFs exhibited normal biophysical behaviours of division, extension and communication in the artificial physiological tissue-like environment.

Based on the 3D culture, HLFs were exposed to NPM-protein corona complexes in a physiological tissue-like environment. Following exposure to specific concentrations of NPM-protein corona, it was expected that the growth and proliferation of 3D-cultured HLFs would be accelerated. However, PM2.5 has been thought to be toxic, which would effectively inhibit cell growth^40,41^. Our results differ from those of previous studies of PM2.5. The protein corona is thought to mitigate the cytotoxicity of 3D-cultured HLFs by reducing the physical interaction of PM2.5 with the cell membrane^42^. In addition to protein corona protection, the discrepancy is most likely due to a difference between a 3D culture and a 2D culture^43^. Numbers of studies have shown that 3D-cultured cells demonstrate higher innate resistance to external stimuli than 2D-cultured cells^44^. For example, evidence shows that the same cytokine inhibits cell proliferation in 2D cultures but promotes cell growth in 3D cultures^45^. In addition, 2D cell models are unable to fully reflect the physiological response in natural tissue-like structures, mainly because the 2D surfaces could result in cell flattening^46^ and cytoskeleton remodelling^47^. Such changes have been shown to alter receptor proteins, drug transporters and metabolizing enzyme activity^48^. These changes always lead to an overestimation of the effects determined from *in vitro* cytotoxic screens. 3D-cultured cells differ morphologically and physiologically from cells in the 2D culture environment^49^. The significant differences between the two culture contexts were manifested in the cell morphology, contraction ability and proliferation rate. Therefore, our results suggest that the 3D HLF culture may mimic the natural *in vivo* setting better than the traditional monolayer (2D) cell culture. The biological response of 3D HLFs to NPM stimulation should be more reflective of *in vivo* cellular behaviour.

In this research, HLF growth was accelerated by stimulation of the NPM-protein corona in the 3D-culture system. However, the addition of an antioxidant was shown to diminish the proliferation efficiency of NPM-protein corona stimulation. This suggests that oxidative stress is associated with HLF proliferation. ROS and GSH are classical biomarkers that reflect oxidative stress. MDA is one of the final products of lipid peroxidation and is responsible for cell membrane damage^50^. 8-OH-dG is the most commonly measured marker of oxidative DNA damage^50^. In our research, these four biomarkers were observed to elucidate the main mechanism of HLF proliferation. Our results show that the NPM-protein corona effectively stimulated ROS generation. Accordingly, GSH depletion was accompanied by ROS generation. However, it did not generate any oxidative damage in the 3D-HLF. Taken together, we concluded that the NPM-protein corona is expected to result in a limited increase in intracellular ROS. ROS are signal molecules that stimulate cell proliferation without inducing oxidative damage. This result is in agreement with the established theory that ROS can generate a specific cellular response, and moderate levels of ROS can stimulate the proliferation of mammalian cells^51,52^. In this research, a single NPM particle showed no efficiency of cell proliferation. NPM-containing metals may impose a negative biological effect against cell growth. However, the NPM-protein corona is proposed to mediate ROS expression by NPM stimulation because the protein coat effectively suppresses the cell membrane binding and cellular uptake of NPM. Accordingly, it reduced intracellular ROS generation and oxidative damage. In addition, the surface charge plays an important role in nanotoxicity. The protein coat may reduce the physical interactions of NPM with the cell membrane, thereby avoiding further oxidative damage to the membrane.

TGF-β1 is a multifunctional set of peptides that control cell proliferation and differentiation^53^. Some studies have shown that TGF-β1 inhibits cell proliferation in 2D cultures^54–56^ but induces 3D-cultured cell proliferation^57^. In this study, TGF-β1 increased ROS production and suppressed GSH, which led to HLF proliferation. These trends were almost the same as those of NPM-protein corona (20 μg/ml)-stimulated HLF proliferation, which suggests that the NPM-protein corona may perform a similar role to that of TGF-β1 in HLF proliferation. In addition, TGF-β1 was also shown to promote cell proliferation by upregulating α-SMA. α-SMA is a key protein expressed by activated HLFs but not by quiescent fibroblasts^58,59^. To reveal the detailed mechanism, α-SMA was measured in all groups of NPM-protein corona preparations. These results indicated that the NPM-protein corona stimulated HLFs to express α-SMA and that α-SMA expression was positively correlated with the NPM exposure concentration. We thus concluded that the NPM-protein corona may generate a limited increase in ROS in HLFs. ROS, as signalling molecules, play an important role in mediating α-SMA upregulation. It is known that pulmonary fibrosis is a consequence of disturbances in the physiologically balanced process of proliferation and apoptosis^60^. Our results demonstrated that nanoscale PM2.5 (NPM) does not necessarily impose direct toxic effects on the human pulmonary system. Nanoscale airborne particles could form a protein corona in the body and initiate HLF proliferation by ROS-mediated α-SMA upregulation, which may further result in pulmonary fibrosis.

In summary, NPM tends to be covered by a protein layer once the particles come in contact with protein. Based on a 3D organotypic cultural model, it was found that the NPM-protein corona stimulated ROS generation but induced no oxidative damage. The protein corona is believed to mitigate NPM cytotoxicity and further stimulate aberrant HLF proliferation. Epidemiological investigations have indicated that human pulmonary fibrosis is associated with PM2.5 exposure in humans. Our results suggest that pulmonary fibrosis may potentially be caused by NPC (NPM-protein corona)-stimulated aberrant HLF proliferation. ROS-activated α-SMA is considered to be one of the pathways of aberrant proliferation. α-SMA is a fibrosis marker that is expressed in fibroblasts. Compared with normal fibroblasts, α-SMA is positively expressed within aberrant proliferated HLFs. In addition, the transformation of fibroblasts to myofibroblasts is also accompanied by the expression of α-SMA. Aberrant proliferation and activation of lung fibroblasts are believed to promote the initiation and progression of human pulmonary fibrosis. Based on biological evaluation via a 3D organotypic cultural model, we found that α-SMA had a positive correlation with the NPM concentration. Meanwhile, ROS-mediated α-SMA expression was positively correlated with the NPM-protein corona concentration. All these results revealed that a nanoscale PM2.5-formed protein corona could promote the aberrant proliferation of HLFs, which may further induce human lung fibrosis.

In this research, the obtained data partly simulated the human responses to NPM involved with lung fibroblasts. 3D-cultured HLFs exhibited more tissue-like properties than those grown in the 2D model. In the future, multiple lung cells should be co-cultured by 3D culture, which could further mimic the physical responses of the human lung. This 3D organotypic culture provides a valuable technology to study the mechanism of PM2.5 involvement in human diseases. These 3D cell culture models are proposed to bridge the gap between 2D cell models and animal models.

## Methods

### NPM collection and characterization

The sampling site was located in Wuhan City, the largest industrial city in central China. The PM2.5 samples were collected on quartz filters by a high-volume PM2.5 sampler (Anderson, USA). Each quartz filter was cut into smaller pieces of approximately 2 cm^2^. These pieces were put into a sterilized beaker with 90 ml of sterilized pure water. A 20 min sonication was carried out below 20 °C. Samples were then filtered using a sterilized filter (<1 μm) to remove larger particles as well as any fibres from the quartz. The collected NPM suspensions were freeze dried in a vacuum for 24 hour and stored at −20 °C. The morphology and size of NPM particles were observed by atomic force microscopy (SPM 3100, Veeco Instruments, Inc., U.S.A.) and scanning electron microscopy (SEM, JSM-6700F, JEOL) at an acceleration voltage of 10 kV. Elements of NPM were analysed by using SEM-EDS (energy-dispersive spectroscopy). Prior to use in experiments, the dried samples were weighed and diluted with a culture medium of HLF.

### Protein corona preparation and characterization

NPM and FBS were mixed, vortexed and placed in an incubator at 37 °C for 24 hour. The mixtures were transferred to a new centrifuge tube and centrifuged at 15000 × g for 20 min at 4 °C. Supernatants were removed, and pellets were dispersed in 1 ml of cold PBS. The collected sample was transferred to a new vial and centrifuged again to pellet the particle-protein complexes. The morphology and size of the particle-protein complexes were observed using scanning electron microscopy (SEM, JSM-6700F, JEOL) at an acceleration voltage of 10 kV. The chemical properties of NPM, FBS and the protein corona were characterized by ATR-FTIR spectroscopy (Nicolet iS 50 FT-IR, Thermo, USA).

### Preparation of the cell exposure solution

NPM was sterilized by ultraviolet light to kill any possible pathogens in the air sample. NPM-protein corona (NPC) samples were prepared as described above and dissolved in the medium by sonication. HLFs (ATCC® PCS-201-013™) were routinely grown using FGM™ fibroblast growth media (Lonza, USA) in a humidified atmosphere of 95% air and 5% CO_2_ at 37 °C. Cells were suspended at 2 × 10^6^ cells/ml and mixed with the NPC solutions. NPM concentrations of NPC-HLFs were fixed at 25, 50 and 100 μg/ml, respectively. To demonstrate the bioactivity of the protein corona, transforming growth factor beta 1 (Sigma, USA), NPC-vitamin C, pure serum and pure nanoscale PM2.5 (NPM) were also mixed with HLFs following the same procedure described above. The final exposure concentrations were 10 ng/ml (TGF-β1), 0.5 mg/ml (vitamin C) and 100 μg/ml (NPM).

### 3D organotypic culture of HLF

Cell exposure solutions were mixed with fibrinogen (10 mg/ml, Sigma, USA) and thrombin (100 U/ml, Sigma, USA) in a ratio of 60:20:19:1 (Note: fibrinogen was dissolved by the HLF medium without FBS; thrombin was dissolved in DPBS containing 0.1% BSA; all mixing was done on ice). Consequently, the final cell exposure concentrations of NPM were 5, 10 and 20 μg/ml. The cell exposure concentrations of TGF-β1 and vitamin C in the 3D systems were 2 ng/ml and 0.1 mg/ml, respectively. Aliquots of these mixtures were seeded in 3D culture systems (15 μl in a microfluidic chip [AIM Biotech, Singapore] and 50 μl in a 96-well plate) for 1 hour at 37 °C. After gelation, the 3D culture models were filled with cell culture medium. The medium was replaced with fresh medium daily.

### Measurement of NPM-stimulated oxidative stress and cell proliferation

Following exposure to the different test groups, HLFs were cultured in the 3D culture systems for three days. All fluorescence images were captured of HLFs cultured in a microfluidic chip. 3D fluorescence images were captured using a confocal microscope (Olympus IX81, USA) and processed with Imaris software (Bitplane Scientific Software). Fluorescent agents were applied to identify ROS (DCFH-DA, Merck KGaA, Germany), GSH (ThioGloTM-1, Merck KGaA, Germany), α-SMA (Abcam, USA), nuclei (DAPI, Invitrogen), and live cells (Invitrogen). The fluorescence intensity of α-SMA expression was analysed using ImageJ software. Other quantitative analyses were performed with 3D-cultured HLFs on cell plates. Prior to testing, the cell medium was removed by pipetting, and 100 µl of gel dissociation saline solution was added (Biovision, USA). The mixtures were incubated at room temperature for 5–10 min and then pipetted up and down with a 1 ml tip until the matrix was dissolved. The plate was centrifuged at 1,000 × g for 5 min at 4 °C, and the supernatant was then removed. Fluorescence intensities (ROS and GSH) were detected using a fluorescence reader (FLx800; BioTek Instruments, Winooski, VT, USA). Cell proliferation was measured using an MTT kit (Nanjing Jiancheng, China). The concentrations of MDA and 8-OHdG were measured using ELISA kits according to the manufacturer’s instructions (Nanjing Jiancheng, China).

### Data availability statement

The datasets generated and/or analysed during the current study are available from the corresponding author upon reasonable request. All data generated or analysed during this study are included in this published article.

### Statistical analysis

GraphPad Prism software was used for statistical analysis of the experimental data and for graphing the results. All data are presented as the means and standard deviation (S.D.). The presence of significant differences among groups was determined by ANOVA. The least significant difference (LSD) method was used to compare the effects between each exposed group and the control. p < 0.05 and p < 0.01 were considered significant.
