# Author Correction: Protein corona of airborne nanoscale PM2.5 induces aberrant proliferation of human lung fibroblasts based on a 3D organotypic culture

**DOI:** 10.1038/s41598-018-25254-6

**Published:** 2018-05-10

**Authors:** Yan Li, Pengcheng Wang, Chuanlin Hu, Kun Wang, Qing Chang, Lieju Liu, Zhenggang Han, Yang Shao, Ying Zhai, Zhengyu Zuo, Michael Mak, Zhiyong Gong, Yang Wu

**Affiliations:** 1 Key Laboratory for Deep Processing of Major Grain and Oil (Wuhan Polytechnic University), Ministry of Education, College of Food Science and Engineering, Wuhan Polytechnic University, Wuhan, 430023 P. R. China; 2 Hubei Key Laboratory for Processing and Transformation of Agricultural Products (Wuhan Polytechnic University), College of Food Science and Engineering, Wuhan Polytechnic University, Wuhan, 430023 P. R. China; 3 Department of Biomedical Engineering, School of Engineering & Applied Science, Yale University, New Haven, 06520 USA; 4 Division of Biostatistics, Department of Population Health, New York University, New York, 10016 USA; 5 Department of Chemical and Biomolecular Engineering, Hong Kong University of Science & Technology, Clear Water Bay, Kowloon, Hong Kong; 6 State Key Laboratory of Silicate Materials for Architectures, Wuhan University of Technology, Wuhan, 430070 P. R. China; 7 School of Biology and Pharmaceutical Engineering, Wuhan Polytechnic University, Wuhan, 430023 P. R. China

Correction to: *Scientific Reports* 10.1038/s41598-018-20445-7, published online 31 January 2018

Michael Mak was omitted from the author list in the original version of this Article. This has been corrected in the PDF and HTML versions of the Article.

The Author Contributions section now reads:

L.Y., M.M., W.Y. and G.Z. conceived and designed the experiments. L.Y., W.P., H.C., C.Q., S.Y., Z.Y. and Z.Z. performed the experiments. L.Y., W.K. and L.L. analysed the data. L.Y., M.M., G.Z. and W.Y. contributed reagents, materials and analysis tools. L.Y., W.Y. and H.Z. took part in the preparation and revision of the manuscript. M.M. provided support for microfluidics and 3D cell culture. All authors have given approval to the final version of the manuscript.

